# ‘It was the deepest level of companionship’: peer-to-peer experience of supporting community-dwelling older people with depression - a qualitative study

**DOI:** 10.1186/s12877-022-03121-4

**Published:** 2022-05-19

**Authors:** Jessica P. S. Tang, Tianyin Liu, Shiyu Lu, C. Y. Sing, Lesley C. Y. Sze, Terry Y. S. Lum, Samson Tse

**Affiliations:** 1 Department of Social Work and Social Administration, The University of Hong Kong, Room 520, 5/F., The Jockey Club Tower, Centennial Campus, Pokfulam Road, Hong Kong, China; 2 Department of Social and Behavioural Sciences, City University of Hong Kong, Hong Kong, China; 3 Sau Po Centre on Aging, The University of Hong Kong, Hong Kong, China

**Keywords:** Peer support, Aging, Mental health, Recovery, Depression, Social ties

## Abstract

**Background:**

There is an ample body of literature examining the experiences and outcomes of peer support services for mental health recovery in western countries. However, formal peer support is only recently adapted and piloted to alleviate depression among older people, and little is known about how the peer-to-peer model might be lived out in the older Chinese population. This qualitative study investigated peer supporters’ (PS) perspectives of their roles and experiences of rendering formal peer support to community-dwelling older adults at risk of or living with depression in Hong Kong.

**Methods:**

The study adopted a qualitative design. Five semi-structured focus groups were conducted with 27 trained peer supporters between ages 54–74 (21 females and 6 males) who had provided peer-to-peer support to older adults at risk of or living with depression in the community for at least 12 months. Thematic analysis was employed to derive content and meanings from the focus group transcripts.

**Results:**

Participants’ mean age was 61.9 years; two-thirds of them were retired and the rest still engaged in part-time or full-time employment. Four themes were identified in relations to the roles and experiences in rendering the peer support services: (1) peerness in health and age-related lived experiences; (2) companionship, social and emotional ties beyond formal support; (3) meaningful roles to facilitate older people’s functional ability; and (4) hopes and actions against the undesirable outcomes of aging. Being a PS might provide meaningful roles for persons in transition to or living in late adulthood, and enable community-dwelling older adults with depression to maintain functional ability. On the other hand, defining the concept of ‘peer’ beyond the shared experience of mental distress, ensuring a healthy boundary between the peers and the service users, maintaining a careful balance between time-limited formal support and stable social ties, and providing self-management training and on-going support appear crucial.

**Conclusions:**

This study of PS’ perspectives and experiences offer insights into the age-specific dimension of the peer relationship. Despite the promising effects it might offer, careful implementation of peer support among older adults is warranted to safeguard against the ensuing loss of meaningful social ties and the potential emotional distress.

**Supplementary Information:**

The online version contains supplementary material available at 10.1186/s12877-022-03121-4.

## Background

Old age is associated with higher risk of common mental disorders [1]. Prevalence of depression peaks in older adulthood [2] as a common late life condition that calls for public health concern. Given the increasing life expectancy across the globe [3], an escalation in the burden of depression on the individuals, their families and the societies are anticipated [4]. Prevention and early intervention are effective for depression in older age and can reduce suffering [5]. Among the non-pharmacological evidence-based interventions, formal peer support has been increasingly adopted to transform mental health services to ensure recovery-oriented care in various parts of the world [6, 7]. The core principles of peer support include recovery-focused, mutual, reciprocal, strengths-based, non-directive, safe, inclusive and progressive [8], while previous research has identified its key components as supportive networks, recovery-orientation, and providing positive role models for others with mental health problems [9]. In addition to role modeling, previous research found building trustful relationships based on lived experience [10] and connecting service users with community resources as the effective mechanisms of peer support [11, 12].

Not only are peer-to-peer support programs beneficial to the service users, they have also been shown to benefit the peer support workers. Over the past two decades, increasing evidence has shown that peer-to-peer support could increase sense of control and self-care, sense of community belongings, satisfaction with life and decreased mental distress among people in recovery [11, 13]. Recent reviews have also recognized the effectiveness of peer support in enhancing service users’ level of hope, feeling of being empowered, quality of life and mental wellbeing [14–16], as well as self-reported recovery, empowerment and social network support [17]. On the other hand, peer support workers also attain a sense of hope and gain skills and knowledge useful in their own situations [18]. Despite the positive outcomes, research has documented the multifaceted challenges and problems that peer support workers face [6, 19], including role conflicts (being a friend vs. paraprofessional to the service users) [20], poorly described role definitions and job structures [21], lack of sufficient and appropriate supervision and support and negative effects on their well-being [22]. Moreover, the notion of ‘peer’ has been narrowly defined as basing on the lived experience of mental illness. To different people, who peer is may vary according to factors such as health condition, age, gender, class and culture [23].

Although much discussion has focused on the outcomes and challenges of peer-based intervention in mental health services, little is known about the roles, and experiences of rendering peer support among the older population. Scattered evidence exists in peer support services for improving mental health, such as reducing depression, increasing quality of life [24], building trustful relationships to effect changes among older adults recovering from depression [25, 26], alleviating loneliness and enhancing resilience [27], and reducing suicidal risk in later life [28]. Previous studies using trained older adult peers and peer volunteers experiencing the same physical problems and issues have been found to be effective in improving physical health, such as the reduction in falls [29], chronic disease self-management [30], alleviation of chronic lower back pain [31] and physical functioning and activities [32].

On the other hand, knowledge about what engaging in formal peer support for community-dwelling older adults with depression would mean to people in their later life remains void. People in late adulthood may face multiple challenges from retirement, decrease in functional abilities, chronic illness and disability, and higher prevalence of grief [33]. There is evidence indicating that complete retirement might lead to increase in difficulties associated with mobility and daily activities, increase in illnesses, and decline in mental health [34]. In addition, a scoping review found that social isolation and loneliness have detrimental effects on physical and mental health in old age [28].

Participation in productive activities, like working part-time post retirement, is found to be beneficial to the alleviation of the adverse health effects [34]. Productive activity is defined as the “production activities of goods and services” that include three main categories: volunteering, employment and caregiving [35]. Previous studies show that volunteering contributes to better health status, prevention of depression and increased quality of life among older adults [36, 37]. With appropriate training, older adults’ engagement in productive activities is also important to address issues related to poorly-resourced professional services [38]. One example is for older adults to engage in mental health services as peer supporters [24]. However, formal peer support in mental health services is a relatively new practice among older adults and in many Asian societies [21]. Western and developed societies still dominate research on peer support services, even though the vast majority of older people, and the rapidly aging populations are in non-western and less developed economies [3]. In Hong Kong, the older population (aged 65 and over) increased continuously between 1988 and 2018 and was projected to rise to 2.44 millions (31.9% of the population) in 2038 while the elder support ratio would decrease [39]. It is imperative to understand how the notion of formal peer support is experienced and perceived in the Chinese community, in particular, among people in late adulthood, to generate insights and knowledge to inform elder service development and practices.

In the present study, we aimed to investigate the peer supporters’ (PS) perspective of their roles and experiences in rendering formal peer support service to community-dwelling older adults recovering from depression through a collaborative stepped-care model - JC JoyAge Holistic Support Project for Elderly Mental Wellness (JC JoyAge) in Hong Kong. The model is a staged approach to the delivery of mental health services, ranging from the least to the most resource-intensive, based on the severity and complexity of the individual’s needs [40] (for details of the service model, refer to [41]. We utilized the focus group method to explore into the meaning of this new role for aging adults.

## Methods

### Design

To develop an in-depth understanding of the notion and experience of peer support among older people in a non-western context, the study adopted a qualitative approach in its design for it would enable deep understanding of subjective experience and social process [42] as well as the meaning attached to these [43]. We utilized the focus group method as it tended to generate discussions among participation through group interactions [44]. Thematic analysis was adopted to identify and to report related themes. Ethical approval to conduct the study was obtained from the Human Research Ethics Committee of the University of Hong Kong  (Approval no.: EA1709021). All methods were performed in accordance with the relevant guidelines and regulations. Written informed consent for study participation, the purpose of the research and the right to withdraw from the study at any time (none withdrew) have been obtained from all the participants.

### Setting and participants

**Fig. 1:**
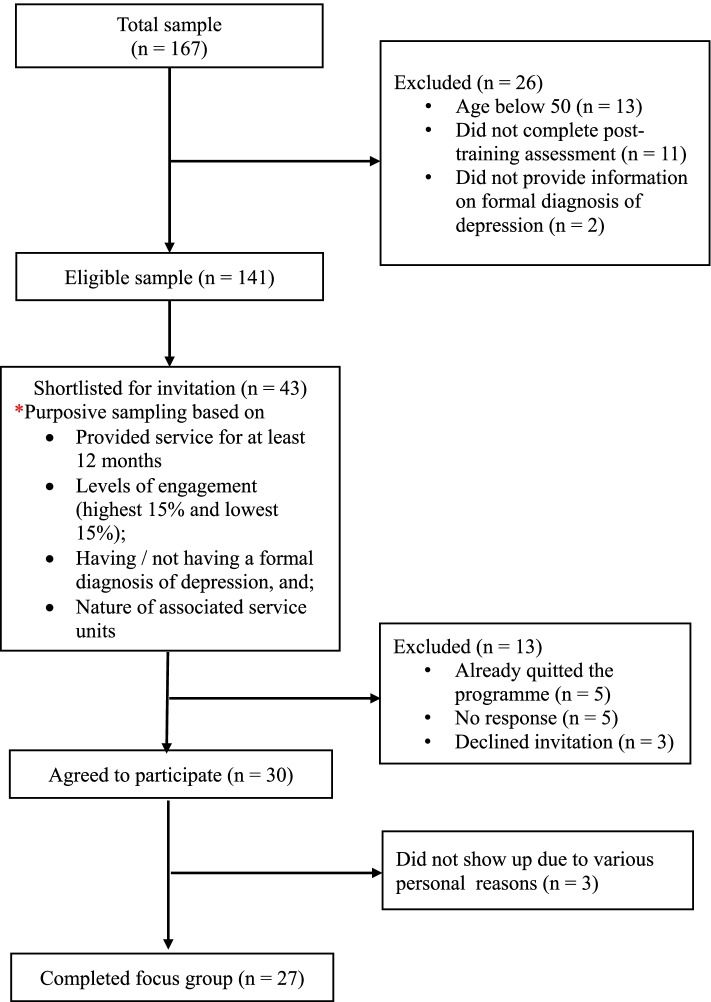
Sample flow chart

JC JoyAge  – was a community-based pilot project that adopted the stepped care and peer-to-peer support models for enhancing the wellbeing of community dwelling older adults with lived experience of depression through the collaboration between community aged care service units and community mental health service units. Participants were recruited from a group of 167 voluntary PS who had provided peer support services to older adults at risk of depression or living with depression in Hong Kong for at least 12 months prior to the current study. The inclusion and exclusion criteria of the service recipients were detailed in the protocol of the service programme [41], and the operational definitions of at risk of or with depression were (a) scored 0–4 on the Patient Health Questionnaire-9 (PHQ-9, [45]) and having risk factors, or scored 5–9; and (b) score 10–14 on the PHQ-9; respectively. The participants in this study were sampled purposively according to the different levels of engagement between October 2017 and September 2018 (high level – 238 to 318 hours; low level – 15 to 56 hours) in peer support services (Fig. 1).

Majority of the PS were retired young-old aged between 60 and 74, with a few retirees in their mid-50s, ranging from having risk factors for depression to those with a diagnosis of depression. They completed a 100-hours certificate training course over 7 months on theories and concepts in older adult depression, mental health recovery, peer support and communication skills, supplemented with experiential learning and practicum. In collaboration with the social workers in the project team, PS engaged in reaching out activities at the individual level: telephone engagement, home visits, initial screening of older persons’ mental health conditions, and support service users in psychoeducation groups and at the community level: street booths and community educational events. The research team sent out invitation letters to recruit PS from four community aged care service units and four community mental health service units in four pilot districts in Hong Kong. A total of 27 PS responded and participated in the study. Participants completed a short questionnaire on demographic information before the focus group discussions. Their average age was 61.9 years (SD = 5.47 years), 21 (77.8%) were female, 6 (22.2%) were male, 14 (51.9%) were recruited from community mental health service units and 13 (48.1%) were recruited from community aged care service units, and 11 (40.7%) reported a history of depression. The average time they spent on volunteer work was 3.8 hours (SD = 2.70) per week. Table 1 summarizes participants’ characteristics and Table 2 summarizes the inclusion and exclusion criteria of clients for peer support service.

**Table 1:** Participants’ characteristics

Items	***n*** = 27	%
Age (Mean, S.D.)	61.9 (5.47)	
Gender		
Female	21	77.8
Male	6	22.2
Service unit		
Community mental health service unit	14	51.9
Community aged care service unit	13	48.1
Employment status		
Retired^a^	18	66.7
Working part-time	5	18.5
Working full-time/ housewife	3	11.1
Unknown	1	3.7
Had previous volunteer experience	17	62.9
Currently involved in other volunteer services	17	62.9
Engagement in volunteer work per week (Hours, S.D.)	3.8 (2.7)	

Notes: ^a^8 years since retirement on average; *S.D.* Standard Deviation

**Table 2:** Inclusion and exclusion criteria of clients for peer support service

Inclusion criteria	Exclusion criteria
(a) Aged 60 years or older	(a) Known history of autism, intellectual disability, schizophrenia-spectrum disorder, bipolar disorder, Parkinson’s disease, or dementia/significant cognitive impairment
(b) At risk of depression or have sub-threshold depressive symptoms based on assessment by social workers	(b) Imminent suicidal risk (temporary exclusion criteria)
(c) Able to give informed consent to participate	(c) Difficulty in communication

### Procedures

The research team carried out five focus group interviews between March and April 2019 in the University of Hong Kong (HKU). Participants were grouped based on their level of engagement hours; three groups (14 participants) were composed of PS with a high level of engagement, and two groups (13 participants) were for those with a low level of engagement. The intention of the groupings was to encourage sharing and minimize peer comparison. Prior to the interviews, participants were informed in writing of the aims and objectives of the focus group, the voluntary nature of their participation, their rights as a participant and the recording arrangements. All participants provided written consent to their participation.

At the start of each focus group, participants agreed on ground rules regarding confidentiality and respect for the views of others. In each focus group, two interviewers co-facilitated the discussion according to a semi-structured interview guide of open-ended questions co-developed by the first author, JT with experience in mental health research and the third author, SL, a social policy researcher. To ensure that the participants could express their views freely, the interviewers were researchers from HKU who did not have any previous encounters with the participants. Participants in the focus groups discussed about their experiences in providing peer support in JC JoyAge, including their perceived roles, enablers and barriers to providing support, tools that they used, meaning and changes that they identified in the peer-to-peer service experiences. Impromptu follow-up questions were also includ ed. Each focus group lasted for 90 to 120 minutes. All five focus groups were audio recorded, and then transcribed verbatim by a research assistant who did not observe the sessions. The transcripts were then checked by CYS for accuracy before the analysis.

### Data analysis and research rigor

Thematic analysis (TA) was used to analyze participants’ narratives generated from the focus groups. TA is a method or identifying, analyzing, and reporting patterns that could provide a rich and detailed, yet complex interpretation of the data [46]. It was believed that the flexibility of TA would allow us to examine and organize our data in a meaningful way for gaining a deep understanding of the role and experience in peer support among older adults. The key researchers (JT, TL, SL and CYS), who were also the interviewers, used the qualitative research software NVivo 12 for organizing the data and conducting the analysis.

The data analysis observed the following steps:

1. Familiarization with the data – listening and reading through the data set.
2. Generating codes to describe the content – reading the transcripts in their entirety and labelling excerpts with initial codes that were relevant to the research questions
3. Identifying themes across different focus groups – going through the codes together one by one according to questions asked in the focus groups to search for significant pattern of meaning, and to collate the codes into potential themes.
4. Reviewing themes – revisiting, reviewing, and refining potential themes against the dataset to ensure distinctiveness and coherence in telling a story that corresponded to the research questions.
5. Defining and naming themes – identifying the focus and scope of each theme; deciding on representative extracts from the dataset and informative names to characterize the essence of the respective theme.
6. Presenting and producing report - systematically reporting the results in relation to the research questions.

The collaborative analytic process included several measures to ensure trustworthiness and credibility: (1) in the initial stage, all members engaged in an intensive discussion to foster reflexivity and co-developed a coding framework from the data from one randomly selected transcript; (2) the research team then worked individually to code the remaining four focus group transcripts based on the coding framework. To provide triangulation in the analysis, the four researchers formed two pairs to cross-examine the coding of the other group, to compare the analysis, and to review data that members did not reach consensus or had omitted; (3) the research team members resolved differences through follow up discussions in subsequent meetings; (4) the findings were presented at project meetings to attain agreement of the emerging results from other project members in JC JoyAge who were not involved in the previous analysis procedures.

## Results

Four key themes emerged from the analysis pertaining to the participants’ experiences: (1) peerness in health and age-related lived experiences; (2) companionship, social and emotional ties beyond formal support; (3) meaningful roles to facilitate older people’s functional ability; and (4) hopes and actions to counter the undesirable outcomes of aging. Nonetheless, participants also encountered psychological struggles while providing support to others despite their meaningful role and positive experiences. There was no significant difference found in the experience between participants with low and high engagement in the service.

### Peerness in health and age-related lived experiences

Sharing personal narratives and lived experience of mental health recovery is a fundamental and crucial component of formal peer support in mental health services for modelling recovery and promoting hope [11, 47]. Although the service was targeted towards older adults with depression, PS seldom disclosed their own lived experience of mental health recovery. Rather, they reported revealing a physical health issue or somatic discomfort as a commonly shared experience at their similar age. Such peerness gave rise to empathy and mutuality in the peer-to-peer relationships.

...some (service users) would say she had bone ache for many years, and so on. Well, because of my age, I have the same experience and I said “yes, me too.”... When she told her adult children about the pain, they could not relate to it, ... I was able to empathize due to our mutual experience, making her feel relieved that I could feel it too. I told her I was relieved as well because the pain I experienced really hurts me too. [PS0003]

Through disclosing similar physical health challenges with service users, PS found themselves helping to alleviate the suffering and sense of isolation that the older adults faced in aging.

... They (service users) were in their 70s to 80s and we (peer supporters) were in our 60s to 70s with (similar) physical conditions like blurred vision, so I would tell them, “yes, I also suffer from blurred vision and have consulted many different doctors...” my sharing of (medical appointment) experience, makes them feel they are not alone (in such suffering), ruminating why other people are not like them. [PS0003]

Despite the differences in personal background, PS identified that they and their service users were equal when it came to physical pain and aging. There was no escape even for health care professionals. Utilizing a first-person testimony, one of the participants normalized the experience of physical pain to support her service user to see that common human vulnerability was a part of life.

My former profession enabled me to support them (service users) sometimes. Meanwhile, I would tell them, “I can see you are suffering from your leg pain. Even though I was working in medical care, I also suffered from the same kind of pain. The poor weather a few days ago triggered my aches. I needed to take medicine to relieve the pain. Physicians and nurses are not exempt from these pains. There are lots of experiences that everyone needs to go through.” [PS0024]

Apart from somatic discomfort, similar backgrounds including age, gender and living in the same neighborhood, also enabled PS to build trustful relationships with service users.

… Since our upbringing was close to that of the service users’, … the living conditions at that time, the neighborhood, and its toughness were similar even though we were like ten years apart in age, we also find some common topics to chat about. If you are not familiar with these common topics, you would find it hard to connect with them, to get them to open up. [PS0006]

These accounts demonstrate that PS have utilized common physical health conditions in aging and other age-related experience to engage and to develop a trustful working relationship with service users.

### Companionship, social and emotional ties beyond formal support

Keeping service users’ company in their daily activities or during hard times was a salient role that participants identified and valued during the 6-9 months peer support service. Participants often revealed the establishment of deep companionship with the service users.

I had a service user who underwent surgery. I sat by his bedside. It was at the deepest level of companionship, the older person found it so hard to let go of me from his life. [PS0016]

Having accompanied the older person to pass through their critical loss in life, some PS were seen as kin or close friends to their service users that went beyond the role of a formal supporter in the care service.

The second time I visited his home, his wife passed away. The funeral was on the Lantern Festival of the lunar new year. I was there. It was important, he had no one around, only me and the social worker from my centre. He felt like I am his relative as my actions touched his heart. Later he told me, ‘If you were not here with me, I have no idea where I would end up, maybe I would go with my wife’. [PS0009]

The significant tie between the PS and the service user created further expectation on the PS that crossed the boundary in the helping relationship.

... we are good friends, he lives in a nursing home now. He asked me to move into the nursing home to live with him. But how can I live with him in the nursing home? [PS0009]

On the other hand, participants also revealed social and emotional ties with service users that surpassed formal peer support. Such strong bonding created psychological struggles during the termination of the time-limited service. PS and service users both experienced uncertainties and separation anxiety towards the “break-up” of the relationship at the end.

We knew about the separation. The social worker made the last visit with us. When it came to the last session, some service users expressed their reluctance, and I told them I couldn’t sleep well the night before. Some asked the social worker, ‘are we going to see them again?’. Some others talked to me, ‘could you get in touch with me some time?’. [PS0007]

I found it very difficult for myself. It was like, how could you leave them (service user) after spending three to four months with them? It was quite difficult. Because of the frequent visits, being with them for a few months, then we need to “break up” when his condition improved. I found it miserable at that moment. [PS0010]

More than one PS reported keeping contacts with their service users after the termination of service. To them, the role boundary in the peer relationship were challenged.

The service period was nine months. When the service comes to an end, what would happen to them? I realized I missed them and worried about them, and there were situations when we still contacted them after termination of the service. [PS0004]

### Meaningful roles to facilitate older people’s functional ability

Participants found meaningful engagement in the role of peer supporters. Some PS saw making contribution to other people’s life as a productive activity that brought about a sense of joy to them.

In my opinion, you shouldn’t stay home and do nothing, living a decadent life. (Peer support) it is not a job, but you get to help the older people, and I feel joyful with meaningful use of time. In other words, you are not sleeping all the time... like [another focus group participant] said, you were helping other people and yourself.... [PS0002]

Moreover, success in building safe and trustful relationships with service users and in alleviating the emotional distress of service users brought valuable roles and a sense of satisfaction to participants. They connected with the joy and happiness of service users.

… We let them know (we) were there to listen, with a direction of ‘prevention (is) better than cure’, preventing them from falling downhill. Preventing the worsening of emotional distress. I think that our presence in this program is very valuable. We were happy when we witnessed their sorrowful faces turning into smiles. Their happiness means our happiness also. [PS0020]

Many PS also saw themselves in the roles of “bridge” to the individuals they supported. They identified their strengths in enabling the socially isolated service users to reach out to the community and in linking them with community resources.

He (service user) might have looked fine and charming in the past, but now he was unwell physically (due to leg pain) and mentally. They might hide themselves at home...We accompany them and bring them back into the community. [PS0004]

I found that, sometimes we could accompany them (service user), or do something for them...because some of them were not aware of the rich community resources. For instance, we linked them up with centres.... [PS0004]

Some PS found themselves as “companions” of the home-bounded service users. They facilitated the older adults to re-activate social activities or desirable health practices, such as going to the market, visiting elderly centers or doing physical exercises. They viewed their engagement in peer support as an important gateway to facilitate the functional ability of the older people recovering from depression in the community.

The intervention groups we had gathered a few older people. The social workers wanted to bring them to the community, for they have been hiding themselves at home most of the time. They hoped to participate but were confined by their physical disabilities, worrying about dizziness and so. That’s why they need a companion. We would accompany them to leave their flat and join the activities. Otherwise, we would do home visits, talk to them, and teach them physical exercises. [PS0018]

Having cultivated a deep understanding about the conditions of the service users, PS served to enable service users in breaking through the negative belief against their poor mobility and to accomplish their unfulfilled will.

He told me he has never been to a tea house, so we went with him, it was joyful. In other cases, their children thought that giving them money and food was good enough and thought of this as caregiving. It was not true, the older people wish to go out for a walk, to feel the community, to feel the world. Their children seldom accompany them, thinking that it’s safer to stay at home because of the (older person’s) poor mobility. When we know them well, learn more about their situations, we take them out to parks, to the seaside...they are very happy. [PS0003]

### Hopes and actions to counter the undesirable outcomes of aging

Participant’s narratives indicated hopes and actions to prevent or to minimize potential undesirable outcomes accompanying aging. Listening to service users’ problems, PS realized that the physical health problems and psychological pain were common human vulnerabilities in aging that might fall on them as well. Such encounters and realization turned into valuable lesson of life to them.

… I think that our service users act like my mirror, reflect my future, I would become them... whether good or bad. On the bright side, I can see their positives, I could be just as good as them. On the negative side, I try not to follow their paths. [PS0025]

The life experience of service users enlightened the PS about changing their own perspectives or taking actions to avoid experiencing similar challenges, such as family conflicts, physical illness and emotional distress that might jeopardize their personal wellbeing in older age.

... (their conditions) deteriorate so quickly and fast. Lots of reasons, very often related to mentality. The body affects mentality, and vice versa. It’s real and observable. We often heard about that but as we visited the older persons more, we witnessed this in person. So I would guard myself and my family from making the same mistakes. Have … learned a lesson from them. [PS0001]

## Discussion

Previous research has identified the key elements of building trust and rapport via shared/ similar lived experiences, role modelling, bridging and engaging within the model of peer support as significant contributors to recovery in mental health care [11, 12, 14, 16]. In this quality study, we examined the experience of a group of trained voluntary peer supporters to aid older people in the community who were at risk of, or living with depression, and the meaning of this new role. We also explored the perceived roles and the effects experienced in the helping process from the PS’ perspective. To our best knowledge, it is the first such study with older peer supporters in a Chinese community. The findings of our study make four key contributions. First, they shed light on the unique expression of ‘peerness’ through physical health and age-related issues among older adults in mental health care and inform a definition of ‘peer’ extending beyond mental distress. Second, they caution against the potential distress created by a conventional time-limited formal peer relationship, especially in a predominant Chinese cultural context. Third, they suggest that peer support may enable aging adults to maintain their functional ability in the community. Lastly, they also unfold the potential and distinctive benefits for people in their later age or retirement life engaging in a new social role (PS to older adults) in preventing the undesirable outcomes of aging.

Our findings are consistent with many previous studies, that sharing lived experience is a key element of peer support [47–49] and is a significant mechanism for building trusting relationships [12, 25]. This sense of peerness is differentiated from befriending the older persons through formally arranged home visits or telephone calls to reduce social isolation and loneliness, with no clear restriction on the befrienders’ profile (e.g., age, interest, and lived experience) [50, 51]. In mental health services, personal narratives of recovery are the predominant experiences that are disclosed, including in depression care [52]. In our study, however, sharing of lived experience was mostly related to physical health problems rather than to depression or other mental distress when it comes to older adults dwelling in the community. This result differed from general peer support model [41], which may be due to the service model and the setting of the current study. The trained peer supporters were working in collaboration with social workers in the community setting, and they shared the tasks to support the clients in their recovery journey. Social workers focused on mental health education and offering psychotherapy, while peer supporters utilised their life experiences and skills to engage and support the older adults enrolled in the stepped care service project. Our findings showed that shared experience of physical pain was common between PS and the older person with whom they worked. Physical or chronic pain is a commonly reported somatic symptom among older persons [53], with prevalence rates up to 55 and 62% at age 60 years and over 75 respectively [54].

Whereas the limited accounts of recovery narratives might be explained by the fact that only 40% of our participants had a formal diagnosis of depression, participants’ tendency to reveal and disclose physical discomfort might also indicate the cultural manifestation of depression among Chinese. Previous research has indicated that Chinese people experiencing depressive mood are more likely to emphasize somatic symptoms than psychological symptoms compared to their western counterparts [55]. Chinese people tend to express their emotional distress through bodily complaints [56]. Social stigmatization of mental disorders might reinforce the tendency not to report mood problems [56] and create barriers to mental health care for community-dwelling older adults [24]. One participant in our study approached the researchers after the focus group and revealed her recovery narrative that she did not want to disclose openly with the group. There is a Chinese idiom “Don’t wash your dirty linen in public” *(jia chou bu chu wai chuan)*, meaning that family shame should be kept within the family. Our findings might suggest that the sharing of lived experience related to physical health problems and age-related functional decline could be a good strategy for PS to build up trust and relationship with older people who experience psychological distress. Yet PS training must also be enriched to include additional module on storytelling to enable older PS to share their recovery narratives for combating the social stigma attached to mental disorders [49].

Our findings show that participants also connected with service users through their closer backgrounds in age, gender and living environment. Such shared backgrounds enabled them to engage with the older person and link the service users with the wider community. These findings suggest that utilizing the lived experience of mental distress alone might be insufficient for establishing formal peer support among older persons as intended. Peer-to-peer relationship among older adults with depression might not simply ride on lived experience of mental distress alone. On the other hand, peerness was more found in shared experiences of health and aged-related conditions, demonstrating the diverse contexts of peerness among them. It is, therefore, important not to limit the definition of peer by the experience of mental distress alone [23] and that matching PS and service users in terms of age, gender and shared local knowledge of living environment is a feasible strategy for effective engagement and establishment of mutuality.

The study also confirmed the unique role of PS in acting as a bridge to alert mental health professionals to service users’ needs and facilitate their engagement with the wider community [12]. Having the PS present to listen to service users recovering from depression, to support them to engage in desired health practices, and to accompany them in reaching out the community like an endearing friend or family member created significant meaning for PS. While affirming that the social ties with the PS served to alleviate isolation and encourage better health practices among service users [57] the strong bonding between them also indicated a companionship and close emotional tie that extended beyond a formal helping relationship.

Despite the benefits of providing peer support, participants’ reports of separation anxiety and psychological struggles at service termination has drawn our attention as to what extend that the introduction of a time-limited peer relationship would be responsive to the circumstances of the older persons. Similar challenges and problems faced by peer support workers were also documented, such as role conflicts (being a friend vs. paraprofessional to the service users) [20] could lead to over involvement, thus demanding a clearer role definition and job structures [21] and appropriate supervision and support [22] to reduce possible stress and burnout for PS. Moreover, old age is a stage in which people face the inevitable loss of close relationships, e.g. spouse and friends [58]. What does establish and terminate a new and positive social tie mean to older people at risk of or living with depression? Should PS maintain a discrete boundary and end contact with service users or can they transform their relationship into an informal one following termination of the formal peer support service? How may providers prepare service users to cope with separation? These questions warrant further examination of applying the intentional peer support model with older adults. To safeguard the mental wellbeing of PS and older adults, the adoption of proactive mechanisms such as Mental Health First Aids and Wellness Recovery Action Plan in the Peer Supporter Training Courses, and the provision a self-management mental wellness programme for older adult service users before the termination of service is recommended. Moreover, human warmth in the interpersonal relationship (*ren qing wei*) is valued by seniors in Chinese culture. Having PS as a provider co-opted in conventional mental health services and one-directional helping relationships [59] might challenge this important value among older Chinese. Given the function of stable social ties on wellbeing among older adults and cultural dynamics, our study prompts a rethink about the constraints of adopting a conventional boundary to the PS/service user relationship in a formal service system and how to foster the transition of support from formal to reciprocal peer relationships in the community.

Participants gained insights from service users’ experiences and exhibited hopes and actions to counter the undesirable outcomes of aging. The increase in awareness and behavioral changes to enhance personal wellbeing affirmed the key elements of reciprocity in peer relationships [8, 23]. In fact, many PS in this study were ‘not there yet’ in the struggles of physical illness and restrictions in mobility. About 60% of them were at risk of depression. Instead of having exchanges on knowledge and past or present recovery experiences, as is common in peer support in mental health, PS in our study utilized the lessons learned from their service users to guard against the foreseeable challenges of aging and risks to wellbeing. Not only does this finding confirm the positive effects of volunteering for productive aging [60], it further suggests that the engagement in formal peer support is a potentially proactive means of promoting healthy aging and preventing mental health problems in old age.

The salient narratives of providing a bridge between socially isolated elders and the community reveal the various abilities and intangible contributions of the older population in combating loneliness [27] and enhancing social connectedness [61]. Getting old does not imply dependence [62]. The active involvement of older PS points to a new social role beyond retirement to promote the functional ability of other elders. The realization of “to be and to do what they have reason to value” [62] regarding healthy aging applies not only to older persons recovering from depression but extends to older PS also. This finding suggests the introduction of peer support in community mental health services for older adults may help to develop social capital, to promote active aging and to maintain the wellbeing of the aging population as outlined in the World Health Organization’s Age Friendly Cities framework [63].

### Strengths and limitations

This study adds to the existing literature on the unique manifestation and role of peer support in mental health care for older adults that warrants a more age-sensitive approach to its implementation. It also offers insight into the promising potential of PS as a meaningful social role for aging adults to counter the negative impact of old age while facilitating the functionality of other community-dwelling elders experiencing depression.

On the other hand, the study investigated the perspectives of PS only and service users’ experiences of listening to the self-disclosure of lived experience were not examined. The data were generated from a cross-sectional design and no distinctive differences were identified between the accounts of participants at risk of and those with lived experience of depression. And due to the design of the intervention programme, the peer supporters in this study went through multiple training phases and were highly selected and self-selected, and their sharing might not be able to be generalised to peer supporter services in other populations and settings. Future research is needed to further investigate and identify significant lived experience (e.g., loss, grief and bereavement), tools, personal competence or life wisdom, and how these would generate meaningful outcomes for older community-dwelling people recovering from depression.

## Conclusions

Peer support in mental health services is a cornerstone to recovery. Yet, it is not a homogenous experience. Lived experiences beyond mental distress and the personal narrative of age-related functional decline could be the common ground to establish and build up trustful relationships with older adults who experience depression to facilitate appropriate responses to the complex predicaments of the older population. Future policy and service planning may develop peer support intervention for older adults as a proactive means of promoting wellbeing and productive aging. Despite its promise, however, formal peer support can be a double-edged sword if insufficient attention is afforded to the loss of social ties during old age. There is a clear need for attaining a deeper understanding of the complexities of peer support and recovery from depression among the older population. Moreover, organizational support and mechanisms such as training, formal supervision and, on-going support to the peer supporters, regular sharing sessions among PS have to be in place to offer a supportive and safe environment for peer support service among older adults.

## Supplementary Information

**Additional file 1.**

## Data Availability

The datasets generated and/or analysed during the current study are available from the corresponding author on reasonable request.
