# Supplementary material for: ‘It was the deepest level of companionship’: peer-to-peer experience of supporting community-dwelling older people with depression - a qualitative study
Source: BMC Geriatr. 2022 May 19;22:443. doi: 10.1186/s12877-022-03121-4 (PMC9121547; doi:10.1186/s12877-022-03121-4)
Supplement: Supplementary file 1 — Additional file 1. [file 12877_2022_3121_MOESM1_ESM.docx]

Supplementary

Table S1. Summary of intervention model and peer supporters’ roles

| Client Group | Criteria | Intervention | Service Unit(s) | PS support |
| --- | --- | --- | --- | --- |
| At Risk | PHQ9 ≤ 4 or with risk factors | Selective prevention: engagement activity + PS | Community aged care | Outreach activities (e.g., booth, telephone survey) |
| Prodromal | 5 ≤ PHQ ≤ 9 without score on item 2 or 6 | Indicated prevention: psycho education + PS | Community aged care |  |
| Mild | 5 ≤ PHQ ≤ 9 & scored on item 2 & 6 | Indicated prevention: group psychotherapy + PS | Community mental health care | Behavioural activation (e.g., company the client in groups) |
| Moderate | 10 ≤ PHQ ≤ 14 | Individual psychotherapy + PS | Community mental health care | Provide personalised support (e.g., regular follow-up, conduct home visits) |
| Moderately severe and above* | PHQ ≥ 15 | Care as usual | Traditional mental health service, HA | N. A |
